# Single-Center Preliminary Experience Treating Endometrial Cancer Patients with Fiducial Markers

**DOI:** 10.3390/life15081218

**Published:** 2025-08-01

**Authors:** Francesca Titone, Eugenia Moretti, Alice Poli, Marika Guernieri, Sarah Bassi, Claudio Foti, Martina Arcieri, Gianluca Vullo, Giuseppe Facondo, Marco Trovò, Pantaleo Greco, Gabriella Macchia, Giuseppe Vizzielli, Stefano Restaino

**Affiliations:** 1 Radiation Oncology Unit, Department of Oncology, “Santa Maria della Misericordia” University Hospital, Azienda Sanitaria Universitaria Friuli Centrale, 33100 Udine, Italy; francesca.titone@asufc.sanita.fvg.it (F.T.); gianluca.vullo@asufc.sanita.fvg.it (G.V.); giuseppe.facondo@asufc.sanita.fvg.it (G.F.); marco.trovo@asufc.sanita.fvg.it (M.T.); 2 Medical Physics Unit, Department of Oncology, “Santa Maria della Misericordia” University Hospital, Azienda Sanitaria Universitaria Friuli Centrale, 33100 Udine, Italy; eugenia.moretti@asufc.sanita.fvg.it (E.M.); marika.guernieri@asufc.sanita.fvg.it (M.G.); sarah.bassi@asufc.sanita.fvg.it (S.B.); claudio.foti@asufc.sanita.fvg.it (C.F.); 3 Clinic of Obstetrics and Gynecology, “Santa Maria della Misericordia” University Hospital, Azienda Sanitaria Universitaria Friuli Centrale, 33100 Udine, Italy; alicepoli94@gmail.com (A.P.); martina.arcieri@asufc.sanita.fvg.it (M.A.); stefano.restaino@asufc.sanita.fvg.it (S.R.); 4 Department of Medicine, University of Udine, 33100 Udine, Italy; 5 Department of Biomedical, Dental, Morphological and Functional Imaging Science, University of Messina, 98125 Messina, Italy; 6 Department of Medical Sciences, Obstetrics and Gynecology Unit, University of Ferrara, 44121 Ferrara, Italy; grcptl@unife.it; 7 Radiation Oncology Unit, Responsible Research Hospital, 86100 Campobasso, Italy; macchiagabriella@gmail.com; 8 PhD School in Biomedical Sciences, Gender Medicine, Child and Women Health, University of Sassari, 07100 Sassari, Italy

**Keywords:** fiducial markers, radiotherapy, endometrial cancer, adjuvant treatment, image-guided radiotherapy

## Abstract

**Purpose**: To present the findings of our preliminary experience using daily image-guided radiotherapy (IGRT) supported by implanted fiducial markers (FMs) in the radiotherapy of the vaginal cuff, in a cohort of post-surgery endometrial cancer patients. **Methods**: Patients with vaginal cuff cancer requiring adjuvant radiation with external beams were enrolled. Five patients underwent radiation therapy targeting the pelvic disease and positive lymph nodes, with doses of 50.4 Gy in twenty-eight fractions and a subsequent stereotactic boost on the vaginal vault at a dose of 5 Gy in a single fraction. One patient was administered 30 Gy in five fractions to the vaginal vault. These patients underwent external beam RT following the implantation of three 0.40 × 10 mm gold fiducial markers (FMs). Our IGRT strategy involved real-time 2D kV image-based monitoring of the fiducial markers during the treatment delivery as a surrogate of the vaginal cuff. To explore the potential role of FMs throughout the treatment process, we analyzed cine movies of the 2D kV-triggered images during delivery, as well as the image registration between pre- and post-treatment CBCT scans and the planning CT (pCT). Each CBCT used to trigger fraction delivery was segmented to define the rectum, bladder, and vaginal cuff. We calculated a standard metric to assess the similarity among the images (Dice index). **Results**: All the patients completed radiotherapy and experienced good tolerance without any reported acute or long-term toxicity. We did not observe any loss of FMs during or before treatment. A total of twenty CBCTs were analyzed across ten fractions. The observed trend showed a relatively emptier bladder compared to the simulation phase, with the bladder filling during the delivery. This resulted in a final median Dice similarity coefficient (DSC) of 0.90, indicating strong performance. The rectum reproducibility revealed greater variability, negatively affecting the quality of the delivery. Only in two patients, FMs showed intrafractional shift > 5 mm, probably associated with considerable rectal volume changes. Target coverage was preserved due to a safe CTV-to-PTV margin (10 mm). **Conclusions**: In our preliminary study, CBCT in combination with the use of fiducial markers to guide the delivery proved to be a feasible method for IGRT both before and during the treatment of post-operative gynecological cancer. In particular, this approach seems to be promising in selected patients to facilitate the use of SBRT instead of BRT (brachytherapy), thanks to margin reduction and adaptive strategies to optimize dose delivery while minimizing toxicity. A larger sample of patients is needed to confirm our results.

## 1. Introduction

Pelvic radiotherapy is administered following hysterectomy and lymph node dissection in gynecologic cancers to decrease recurrence rates and enhance progression-free survival [1,2,3]. Additionally, it can be used as a definitive treatment for locally advanced cervical cancer, often combined with concurrent chemotherapy whenever possible [4]. Intensity-modulated radiotherapy (IMRT) minimizes radiation exposure to healthy tissues, including the small bowel, rectum, and pelvic bone marrow, thereby reducing short-term and long-term side effects. However, ensuring accurate delivery with these precise techniques necessitates careful consideration of organ motion, setup variations, and delineation errors to prevent positional inaccuracies. Indeed, several studies have shown that interfractional vaginal motion (IVM) occurs during radiotherapy, particularly in post-operative gynecological patients, due to natural variations in bladder filling and rectal voiding [5,6,7]. Daily image-guided radiotherapy (IGRT) using fiducial markers (FMs) is proposed as a technique to address interfractional vaginal motion [5,8], with FMs serving as surrogates to determine the position of the vaginal–parametrial clinical target volume (CTV) [9]. This paper presents a case series of patients who underwent postoperative radiotherapy for endometrial cancer. In this cohort, we conducted an observational descriptive study to assess the role of FMs in guiding radiation therapy within the gynecological setting. FMs are clinically used to visually validate the position of the vagina in the PTV. The goal was to emulate the consolidated paradigm of radiation treatment of the prostate, where the use of FMs is common [10].

## 2. Methods

### 2.1. Clinical Cases

We present a case series comprising six patients who received radiotherapy (RT) at our institution from April 2022 to February 2024. These patients underwent RT after implanting 0.40 × 10 mm gold fiducial markers (Gold Anchor™ Naslund Medical AB, Huddinge, Sweden). The patients were treated with adjuvant locoregional radiotherapy after hysterectomy for gynecological cancer. They were diagnosed with endometrial cancer and underwent surgery followed by adjuvant radiotherapy. The six patients diagnosed with endometrial cancer underwent procedures according to the latest guidelines [1], which included hysterectomy, bilateral salpingo-oophorectomy, sentinel lymph node (SLN) mapping and excision, or pelvic lymphadenectomy. These patients were classified into intermediate-, intermediate-high-, and high-risk groups. Following multidisciplinary discussions, they were considered eligible for adjuvant RT. Five patients (pt1, pt2, pt3, pt5, pt6) underwent radiation therapy targeting the pelvic disease and positive lymph nodes, with doses of 50.4 Gy in twenty-eight fractions and a subsequent stereotactic boost on the vaginal vault at a dose of 5 Gy in a single fraction. One patient was administered 30 Gy in five fractions to the vaginal vault (pt4). Due to significant comorbidities, body habitus, and anatomical limits of our group of patients (median age, 77.8 years; median BMI, 28.8), we chose to adopt a stereotactic approach considering its potential ability to deliver high radiation doses, mimicking the BRT dose distribution [11].

### 2.2. Procedural Method: Insertion of Markers

After providing informed written consent, the FM implantation procedure was performed by a gynecologist in an outpatient setting, with the patient in a gynecological position. Before the procedure, local anesthesia was used with the application of lidocaine spray on the vaginal cuff, and a transvaginal ultrasound was performed for proper evaluation of the target area. FMs were inserted in the vaginal cuff with the following steps: the first marker was placed on the bevel of a 20-gauge × 20 cm injection needle; the needle was inserted into the target area; finally, the marker was released at this level. The number of markers inserted was three. The configuration of the three fiducial markers was designed to enable triangulation and positional measurement in different planes, serving as a surrogate for the vaginal cuff volume. At the end of the procedure, the patient did not report pain or discomfort. No antibiotic prophylaxis was required, nor was cessation of anticoagulant/antiplatelet medications.

### 2.3. CT Simulation and Planning

For the five patients with intermediate-high risk who also received pelvic irradiation, the implantation was executed just after the last fraction of the pelvic treatment. For the patient with five-fraction treatment focused on the vaginal vault, FMs were inserted ca. 48 h before the delivery of the treatment. The day after the insertion, the patient performed a planning CT (pCT).

For all the cases, before the simulation, patients were advised to ensure their bladder contained approximately 200–300 mL of urine and to empty their rectum. This protocol has two potential advantages for the quality of the treatment: sparing of the rectum and the bladder, reducing the vaginal motion [7]. The pCT was acquired with the patient in the supine position, headfirst to the gantry, immobilized with a Combifix™ frame (Civco Inc.^®^, Coralville, IA, USA). The scan parameters were 2 mm slice thickness and 120 kV as the kV voltage. Images were then transferred to a deep learning-based AutoContouring Platform (Limbus AI 1.7, Radformation, New York, NY, USA) and subsequently automatically imported with its associated structures into an Eclipse 18.0 Treatment Planning System (TPS) (Varian Siemens Healthineers, Zug, Switzerland). A radiation oncologist (RO) experienced in gynecological settings supervised the organs at risk (OARs) and delineated the vaginal CTV (clinical volume target) according to the consensus guidelines, with the guidance of the FMs, too [12]. The PTV was obtained by expanding the CTV by 10 mm in three dimensions.

This margin takes into account the median organ motion in the majority of our gynecologic patients undergoing postoperative pelvic CBCT-guided RT.

Using Eclipse TPS for the True Beam Radiotherapy System (Varian Siemens Healthineers) equipped with an HD (high-definition) MLC (multileaf collimator), 6FFF (flattening filter-free) plans based on the VMAT (volumetric modulated arc therapy) technique were generated. Two full arcs were planned with the collimator rotated so as to minimize the tongue-and-grove effect and interleaf leakage. The first arc was created in the clockwise direction, the second—in the counterclockwise direction. Using Eclipse tools, we identified the centroid of each FM.

The planning goals were to ensure that at least 95% of the PTV received the prescribed dose, with the maximum dose (up to 0.03 cc) not exceeding 107% of the prescribed dose. Dose summation in terms of EQD2 (equivalent dose in 2 Gy per fraction) of the boost plan with the pelvic plan was performed via Velocity^TM^ (Varian Siemens Healthineers) to generate a composite dose received by the rectum and the bladder inclusive of the different dose prescription schedules. For all the plan sums, we obtained compliance with the Embrace protocol constraints. [13].

### 2.4. IGRT and Delivery

For all the patients, the preparation of the rectum and the bladder was a prerequisite before each treatment fraction, similar to the preparation prior to the CT simulation phase. After the initial setup of the patient by the radiation therapist (RTT), a 2 mm CBCT was acquired.

The recent iterative image reconstruction algorithm iCBCT (Varian Siemens Healthineers) was used to enhance the overall image quality. Unlike conventional CBCT, which suffers from reduced image quality due to increased radiation scatter, iCBCT improves performance by utilizing scatter correction and statistical reconstruction, resulting in higher-quality images. [14].

Six degrees of freedom (6-DoF) registration with the pCT was executed. When the rotation deviations were greater than 1°, the radiation therapist’s team corrected the patient setup to reduce the discrepancy. A well-experienced RO carefully analyzed the match based on bony anatomy and FMs to confirm the patient’s setup, monitor the bladder/rectum filling states, and visualize the target. The RO focused on the correspondence of the FMs that compensate for the limited quality of CBCT imaging.

If the filling of the rectum and the bladder was not adequate, the patient was asked to drink according to the protocol and/or empty her bowel with the help of an enema; then, CBCT was repeated. In this manuscript, the CBCT that guides the decision of delivering the fraction is named triggering CBCT (tCBCT), while the CBCT acquired at the end of the second arc is labeled final CBCT (fCBCT).

Using the on-board imaging (OBI) technology and the advanced IGRT & Motion package (Varian Siemens) available on the Truebeam platform (v2.7), a two-dimensional (2D) real-time fiducial marker position verification technique was adopted as an online IGRT strategy. The application enables planar kV images to be acquired during arc delivery using an orthogonal on-board imager (OBI). In more detail, the proprietary software automatically detects markers using a search region represented by a 10 mm diameter circle around the centroid of each marker on each 2D kV image acquired every 3 s during arc delivery. The system provides valuable visual feedback on the distance of the absolute FM position from the expected one represented during the delivery: the circle and the crosshair are shown in green and red colors, respectively, to indicate that the fiducial marker is within or out of the circle diameter that represents our operative tolerance.

Considering the safe CTV–PTV margin adopted, the FMs’ trajectory was only observed during plan delivery, without stopping the irradiation in the case of significant shifts of FMs. At the end of the fraction delivery, CBCT was repeated and compared with the pCT to monitor the rectum and the bladder.

### 2.5. Data Reporting

To investigate the operational role of FMs in the entire treatment procedure, we collected and analyzed the cine movies of the 2D kV-triggered images during the delivery and the image registration between the tCBCTs and fCBCTs and the pCT. The overall motion of the FMs was quantified by the displacement of the center-of-mass position of the marker configuration.

Retrospectively, every tCBCT and fCBCT was contoured in terms of the rectum and the bladder by Limbus AI (release 1.8, RadFormation). The same skilled RO who delineated the original volumes of interest supervised this auto-segmentation process and contoured the vaginal vault (CTV) relying on the FMs. The three volumes (rectum, bladder, and CTV) obtained were compared to the original ones delineated onto the pCT: for every pair of volumes, the Dice similarity coefficient (DSC) was determined.

Based on the American Association of Physicists in Medicine Task Group 132 (TG-132) report [15], contours presenting a DSC < 0.8 are considered inaccurate. However, considering the quality of CBCT imaging compared to the pCT’s one, a threshold value of 0.7 was chosen from the literature on DSC-based segmentation validation [16,17].

For the vaginal vault, the DSC was also calculated between the relative volume delineated onto tCBCTs and fCBCTs to quantify the effective motion of the target.

## 3. Results

From April 2022 to February 2024, six gynecological patients underwent radiotherapy with implanted FMs on the vaginal cuff. The patients’ characteristics, histology, risk group, and type of surgery are detailed in Table 1.

The implementation of FMs, which enhance the accuracy of radiotherapy, was associated with positive outcomes regarding the quality of life of the patients in our case series. The results indicate that after the implantation of fiducial markers, the patients maintained a good quality of life and did not report any significant negative impact. At the 1-year follow-up, all patients had completed radiotherapy and demonstrated optimal tolerance, with no reports of acute or long-term toxicity.

**Figure 1:**
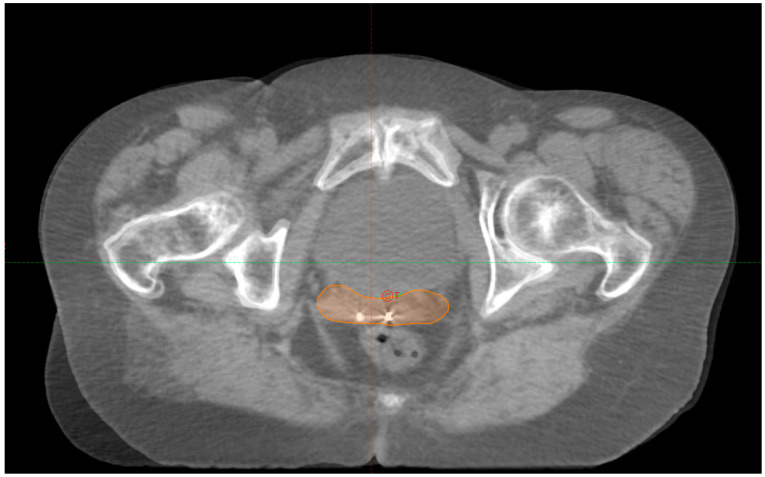
An example of the IGRT technique applied in this observational study (pt4—fraction 2); pCT imaging with the vaginal CTV segmented registered with daily CBCT based on the alignment of the implanted FMs.

We did not observe any loss of FMs during or before treatment. A total of twenty CBCTs were analyzed across ten fractions (in Figure 1, there is an example of match pCT-CBCT). In one patient, the treatment delivery was postponed to the following day because of an unsatisfactory preparation level of the rectum.

The median rectum volume on pCT was 44.5 cc (range: 36.2–53.6 cc), while the median volume of the rectum on tCBCT was 48.9 cc (range: 41.3–56.2 cc), and the corresponding value for fCBCT was 50.9 cc (range: 41.7–74.9 cc). The median bladder volume on pCT was 304.3 cc (range: 193–352 cc), while the median volume of the bladder on tCBCT was 386.2 cc (range: 130.4–484.2 cc), and the corresponding value for fCBCT was 444 cc (range: 146.2–540.6 cc).

The median CTV on pCT was 33.8 cc (range: 22.0–39.9 cc), while the median volume on tCBCT was 32.8 cc (range: 21.0–38.4 cc), and the corresponding value for fCBCT was 33.2 cc (range: 22.5–39.3 cc).

Dealing with the similarity index (DSC), Table 2 summarizes the distributions of DSC values for all the patients and combinations (tCBCT vs. pCT, fCBCT vs. pCT, and fCBCT vs. tCBCT) for the bladder, rectum, and vaginal vault (CTV). The median DSC value for the bladder was 0.76 (0.72–0.92), 0.79 (0.64–0.92), and 0.90 (0.74–0.96) for tCBCT vs. pCT, fCBCT vs. pCT, and fCBCT vs. tCBCT, respectively. The median DSC value for the rectum was 0.72 (0.58–0.81), 0.69 (0.51–0.82), and 0.82 (0.56–0.90) for tCBCT vs. pCT, fCBCT vs. pCT, and fCBCT vs. tCBCT, respectively. Lastly, the median DSC value of the CTV was 0.72 (0.63–0.80), 0.71 (0.20–0.79), and 0.81 (0.18–0.92) for tCBCT vs. pCT, fCBCT vs. pCT, and fCBCT vs. tCBCT, respectively.

Registration revealed mean setup errors for the anterior–posterior direction, the superior–inferior direction, and the lateral direction were 2.0 ± 4.4 mm, 0.9 ± 3.2 mm, and −0.2 ± 4.4 mm, respectively.

**Figure 2:**
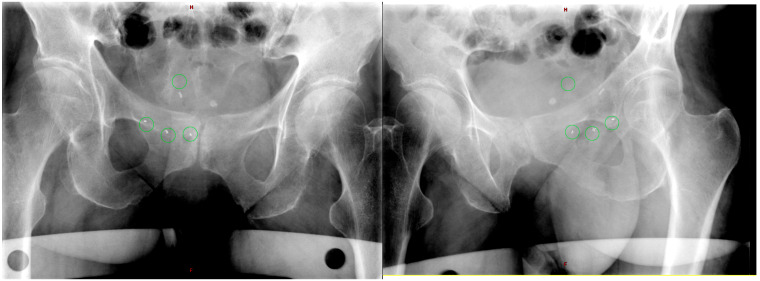
Two frames of the real-time target tracking system from our database (first arc for fraction 2 of pt4). The on-board proprietary search algorithm finds the locations of the fiducial markers in each triggered kV image and determines if they are within a predetermined tolerance from the expected location. In the images, the software erroneously identified a fourth circle, significantly far from the region of interest, probably due to the presence of calcification. Accordingly, it was not considered.

Regarding the results of FM-based tracking (Figure 2), we collected data from ca. 500 frames. In general, we did not observe deviations from the control ROI, except for three fractions with two patients. The most critical patient was pt4. In two out of five fractions of her treatment, FMs shifted outside the 5 mm tolerance level: the second fraction with a mean distance value from the FM’s centroid of 7 mm and the fourth fraction that presented major deviations (mean value: 8.5 mm). In both cases, we registered the out-of-tolerance condition approximately halfway along the second arc. Considering our observational approach guaranteed by a safe CTV–PTV margin, we did not stop the delivery. We attributed these FM shifts to an important and sudden change in the status of rectal filling. The fraction number three was quite critical, too: the mean distance of the FM’s centroid from the center of the ROI was ca. 5 mm, so judged borderline. Probably, the patient would have benefited from the use of smaller CTV–PTV margins and an adaptive strategy during beam delivery (pausing the irradiation and correcting the OARs’ conditions and/or eventually re-adjusting the setup).

Furthermore, the single treatment fraction of pt6 showed a similar entity of motion of FMs (mean value: 6.5 mm), always in the second part of the plan delivery.

Despite using a strict filling protocol to minimize variations in bladder and rectal volumes before the fraction delivery, substantial changes were observed in some patients, as one might expect in this category of patients (frail elderly postsurgical patients). However, the median value of DSC scores for the bladder and the rectum in the CBCT that guides the delivery was overall acceptable (>0.70), showing a generally sufficient agreement with the planning reference values. The observed trend showed a relatively emptier bladder compared to the simulation phase, while the bladder filled during the delivery. This resulted in a final median Dice similarity coefficient (DSC) of 0.90, indicating strong performance. More critical was the impact of rectal reproducibility; further, due to its size, it showed wider discrepancies and mostly intrafractional variability due to rectal contents such as feces and gas. The DSC values for the vaginal vault worsened during the planned irradiation, probably because of the rectal changes. The more problematic fractions were the second and the fourth fractions of pt4 and the fraction of pt6, which are exactly the same fractions where significant contextual FM shifts were observed. On the other hand, the target coverage was assured, as already commented, by having adopted a sufficiently large CTV–PTV margin.

## 4. Discussion

There are several sources of uncertainty in the gynecological setting, but interfractional and intrafractional variability of the internal pelvic anatomy compared to the simulation setup are probably the most important ones. In particular, intrafractional and interfractional vaginal movement during beam delivery merits special attention as demonstrated by numerous studies [7,18]. Daily image-guided radiotherapy (IGRT) with fiducial markers implanted in the vaginal cuff has been proposed as a method to address interfractional vaginal motion. The presence of FMs can indeed aid in the identification of the radiation target during treatment, facilitating intrafractional monitoring.

In this case series, we describe our preliminary daily IGRT experience in a pool of six patients affected by endometrial cancer who received postoperative radiotherapy after the implantation of gold FMs in the vaginal vault. The FMs in the vaginal setting helped us to visually validate the position of the vagina in the target region, compensating for the poor quality of soft tissue imaging in the CBCT. The Varian Advanced IGRT & Motion Package enabled planar kV images to be acquired during treatment delivery, permitting real-time control of the target movement. The goal was to replicate the paradigm used in the radiotherapy of localized prostate cancer, where fiducial markers (FMs) serve as surrogates for the prostate gland, guiding the process from segmentation on simulation images to the delivery phase. Indeed, our institution has extensive experience in adopting highly hypofractionated treatment regimens since 2011 [18,19,20].

The markers’ implantation was a quick and safe procedure, not requiring any hospitalization, with limited discomfort for the patient, and without significant FM loss or migration, in line with previous reporting [21]. To date, few studies on this issue are available. Some studies have reported that variations in the rectal or bladder volumes could be correlated with significant displacement of the vagina, causing changes in the target coverage or OAR doses [17,22,23]. Jurgenliemk-Schulz et al. reported that a 100 cc difference in rectal volume resulted in a 1 cm vaginal shift in the anterior–inferior direction [24]. Harris et al. also showed that the interfractional vaginal motion had a considerable range (0.60–20.2 mm) [25]. The conclusions of these papers focused on the correlation between the target motion and the variability of the OARs, as well as their impact on delivery time.

Moreover, Jhingran et al. estimated changes in the vaginal apex position (mainly in the anterior–posterior direction) as a function of rectal and bladder filling [6]. Rash et al. analyzed one hundred and forty-five daily CBCTs from a set of five patients with gold FMs implanted in the vaginal vault, observing important deviations (in one patient, the FM displacement was outside the PTV in ca. 16% of fractions) [23]. Monroe et al. described the two-year clinical results of twenty-six patients monitored with IGRT with FMs, demonstrating notable cross-border changes that needed a median correction of ca. 1 cm. At two years, they observed no recurrence and a low frequency of acute GI/genitourinary (GU) toxicity [8]. Recently, Buijs et al. presented an ART strategy based on adapting the average interfractional vaginal motion during the initial treatment fractions with the intent to significantly reduce the CTV–PTV margins in postoperative gynecological tumors [5]. In our experience, the controlled bladder filling and rectal voiding confirmed their fundamental role in the consistency of the treatment delivery. The combination of these features allows the potential for the most significant reduction of the clinical margins: the reduction of the CTV–PTV margins implies a decrease in the irradiated volumes and a possible reduction in the OAR doses. This potential advantage paves the way for an SBRT approach in patients unable to receive a brachytherapy procedure. Based on the analysis of the collected data, a threshold of 5 mm in the cranio-caudal (CC) direction and 4 to 5 mm in the anteroposterior (AP) direction appears to be an effective set of values when combined with the triggering kV imaging approach. Under this method, beam delivery is initiated and continues until two out of three fiducial markers fall within the allowable range. If the markers do not fall within the allowable range, treatment is paused, and additional 3D imaging is performed. After adjusting the couch, treatment can resume. However, if significant variations in the conditions of the organs at risk are observed, appropriate actions must be taken regarding the patient. Therefore, the use of fiducial markers assists radiation therapy technologists in performing intrafractional monitoring of treatment delivery, enabling the identification of potential interruptions in the treatment process when necessary. This study is biased by a small sample size and a short follow-up period to assess long-term radiotherapy toxicity. However, it was conducted at a single institution by the same group of highly experienced operators in managing these types of cancers. Further research is necessary to explore potential critical issues, such as fiducial marker migration or their role in multifractionated treatments involving pelvic lymph nodes.

## 5. Conclusions

Our experience has permitted the safety of patients with gynecological cancer who underwent an insertion procedure of a set of three FMs. The study showed evidence of fiducial markers’ visibility, thus providing a more practical and robust registration of CBCT to pCT.

Daily IGRT with CBCT, aligned with the use of FMs implanted in the vaginal cuff, presented a viable method of IGRT before and during the treatment of postoperative gynecological cancer to compensate for the inter- and intrafractional vaginal motion.

While acknowledging the limited sample size of our observational investigation, our preliminary results appear to confirm the adoption of an “adaptive” strategy in the vaginal setting, combining a significant reduction of CTV–PTV margins (isotropic 5 mm) and an active kV-based tracking during the arc delivery.

This approach, along with precise patient preparation, may enable the adoption of SBRT for elderly patients and those with anatomical characteristics that make them ineligible for traditional intracavitary BRT, improving previous literature experiences [26,27]. This technique would avoid dose reduction or target misses while potentially decreasing the risk of toxicity. Our limited cohort primarily consisted of older patients (mean age: 77.8 years) unable to receive brachytherapy treatment. Future investigations should stratify results by age, body habitus, and anatomical features to assess the broader applicability of this IGRT approach in the adjuvant endometrial setting.

## Figures and Tables

**Table 1:** Endometrial cancer patients.

Endometrial Cancer Patients	N
No. of patients	6
Mean BMI (range)	28.8 (23–33.5)
Mean age (range)	77.8 (68–87)
FIGO Ib	2
FIGO II	3
FIGO IIIa	1
**Grading**	
Grade 1	3
Grade 2	3
**Type of LVSI**	
LVSI+	2
LVSI−	4
**Risk group**	
Intermediate	2
Intermediate-high	3
High	1
**Lymph node removal**	
SNL	2
Pelvic lymphadenectomy	3
Nx	1
**Treatment**	
**Adjuvant RT**	6
**Radiotherapy toxicity**	
Acute toxicity	0
Long-term toxicity	0
**Patient’s status**	
NED	6

**Table 2:** Summary of the Dice coefficients for the bladder/rectum/CTV (vaginal vault).

DSC	Bladder			Rectum			Vaginal Vault		
	tCBCT vs. pCT	fCBCT vs. pCT	tCBCT vs. fCBCT	tCBCT vs. pCT	fCBCT vs. pCT	tCBCT vs. fCBCT	tCBCT vs. pCT	fCBCT vs. pCT	tCBCT vs. fCBCT
pt1_singlefx	0.92	0.92	0.92	0.81	0.81	0.81	0.80	0.79	0.83
pt2_singlefx	0.88	0.87	0.93	0.58	0.62	0.80	0.75	0.77	0.81
pt3_singlefx	0.76	0.88	0.83	0.73	0.71	0.82	0.79	0.72	0.83
pt4_f1	0.75	0.72	0.88	0.72	0.64	0.83	0.72	0.61	0.64
pt4_f2	0.72	0.68	0.93	0.68	0.66	0.74	0.63	0.43	0.48
pt4_f3	0.75	0.64	0.94	0.71	0.51	0.86	0.71	0.70	0.81
pt4_f4	0.75	0.73	0.86	0.69	0.78	0.56	0.68	0.20	0.18
pt4_f5	0.75	0.78	0.87	0.69	0.78	0.87	0.66	0.73	0.92
pt5_singlefx	0.78	0.80	0.96	0.78	0.82	0.90	0.65	0.64	0.82
pt6_singlefx	0.84	0.85	0.74	0.73	0.53	0.58	0.74	0.71	0.46

## Data Availability

The data that support the findings of this study are available from the corresponding author upon reasonable request.
